# Low-Concentration Sucrose Intake During Puberty Alters Cholesterol Metabolism and Circulating Testosterone Levels in Male Mice

**DOI:** 10.3390/nu18213481

**Published:** 2026-10-23

**Authors:** Aya Fujii, Yukihiro Yoshimura

**Affiliations:** Graduate School of Food and Medicinal Sciences, Kobe Gakuin University, 518 Arise, Ikawadani-cho, Nishi-ku, Kobe City 651-2180, Japan; f9zgfd01@s.kobegakuin.ac.jp

**Keywords:** sucrose, puberty, cholesterol, steroidogenesis, testosterone, non-HDL-cholesterol

## Abstract

**Background:** Pubertal sucrose intake is a public health concern, but its effects on cholesterol metabolism and male reproductive endocrinology remain poorly understood. Rodent studies of this window have generally used 30% (w/v) sucrose or 20% (w/v) fructose solutions; beverage-like concentrations have been examined less often. Reported effects of dietary sugar on circulating testosterone are inconsistent; we hypothesized that differences in developmental stage might account for this discrepancy. **Methods:** Male ICR (Institute of Cancer Research) mice had free access to a 10% (w/v) sucrose solution from 4 to 8 weeks of age. We assessed hepatic and plasma lipids, testicular cholesterol and testosterone, hepatic acetyl-CoA carboxylase (ACC) and AMPK phosphorylation, and expression of cholesterol metabolism- and steroidogenesis-related genes. All measurements were performed after a 16 h fast, during which the sucrose solution was replaced with water. **Results:** Sucrose selectively elevated hepatic and plasma cholesterol without altering triglycerides, the circulating increase being confined to the non-high-density lipoprotein fraction. Phosphorylation of ACC1 and ACC2 rose while AMPK phosphorylation was unchanged. Peritesticular fat weight increased despite unchanged body weight and energy intake. In the testes, cholesterol content and several steroidogenic transcripts increased, whereas steroidogenic enzyme protein was unchanged. Plasma testosterone increased, whereas intratesticular testosterone did not. **Conclusions:** In male ICR mice, a 10% (w/v) sucrose solution during puberty supplied approximately 25% of total energy intake, with a comparable fall in solid-chow intake; it was associated with a cholesterol-predominant rather than triglyceride-predominant lipid response and higher circulating testosterone. Testicular cholesterol content and plasma testosterone were both higher while steroidogenic enzyme protein was unchanged, indicating an association between the two rather than a change in steroidogenic capacity.

## 1. Introduction

The excessive consumption of added sugars, particularly sucrose and high-fructose corn syrup delivered through sugar-sweetened beverages (SSB), is a major public health concern worldwide [1]. Children and adolescents are among the highest consumers of added sugars, with SSB representing their leading dietary source [2,3]. Puberty is a critical developmental window that is characterized by rapid somatic growth and maturation of the hypothalamic–pituitary–gonadal axis that establishes adult reproductive capacity [4]. During this period, nutrition has an important influence on development [5]. Despite the high prevalence of SSB consumption among children and adolescents, whether habitual sugar intake during puberty affects the trajectory of reproductive hormone production remains unclear.

Sucrose is hydrolyzed to glucose and fructose upon ingestion. Although glucose is metabolized systemically, fructose is primarily metabolized in the liver, where its unregulated entry into glycolysis promotes hepatic de novo lipogenesis (DNL) [6,7]. This has been characterized largely as a triglyceride (TG)-predominant response in adult models at relatively high-fructose loads, with rodent studies using 10–20% fructose solutions reporting elevated circulating TGs [8,9,10], whereas lower concentrations have not necessarily reproduced these changes [11]. This dose-dependence raises the question of whether sugar intake during puberty, when the fructose load delivered by typical SSB consumption is comparatively modest, elicits the same hepatic lipid response as that in adults.

In addition to TG metabolism, fructose and sucrose intake may influence cholesterol (Chol) homeostasis, which is particularly relevant during puberty because Chol is an obligatory substrate for steroidogenesis, including testicular testosterone biosynthesis [12]. Steroidogenic cells acquire Chol through endogenous synthesis and receptor-mediated uptake of circulating lipoproteins, with the relative reliance on these routes differing among steroidogenic organs and across species [13]. Puberty is accompanied by a marked increase in Leydig cell steroidogenic activity and androgen production [14], which increases the demand for cholesterol as a biosynthetic substrate. As sucrose solution intake exacerbates allergic contact dermatitis in rats [15], its effects are not confined to lipid metabolism. Together, these observations raise the possibility that sucrose intake during puberty, a period of both heightened Chol demand and increasing dietary sugar exposure, could influence testicular Chol acquisition and, in turn, testosterone production; however, this possibility has not been directly tested.

Notably, the reported effects of sucrose and fructose intakes on circulating testosterone concentrations have been inconsistent across studies. Rodent studies in which dietary sugar exposure was confined to the juvenile–peripubertal window revealed elevated testosterone levels [16,17], whereas exposure extending into adulthood was associated with suppressed testosterone levels [18,19]. As these studies differed considerably in the developmental stage during which sugar exposure occurred, we hypothesized that the timing of exposure, rather than sugar intake, might account for this discrepancy. Given the concurrent increase in the Chol demand for pubertal steroidogenesis, we hypothesized that sucrose intake during this window would increase, rather than decrease, circulating testosterone concentrations.

In this study, we aimed to test the hypothesis that sucrose intake during puberty (4–8 weeks of age) increases circulating testosterone levels in male mice. We examined the effects of a 10% (w/v) sucrose solution—one-third the concentration of the sucrose solutions used in the two most closely comparable studies [16,19], and at the concentration of a commercial sugar-sweetened beverage, although the sucrose consumed still supplied approximately 25% of total energy intake—on hepatic and plasma lipid profiles, testicular Chol metabolism, steroidogenic gene expression, and testicular Chol and testosterone concentrations in male ICR (Institute of Cancer Research) mice. Even with this more dilute solution, sucrose intake elicited a Chol-predominant rather than a TG-predominant lipid response and increased circulating testosterone levels, which is consistent with our hypothesis.

## 2. Materials and Methods

### 2.1. Animals

Male ICR (Institute of Cancer Research) mice, stock designation Slc:ICR, 3 weeks of age, were purchased from Japan SLC, Inc. (Shizuoka, Japan) and housed in a conventional animal care facility at Kobe Gakuin University, Japan. ICR is a closed-colony outbred stock and, unlike an inbred strain, is not genetically defined [20]; outbred stocks nonetheless do not show greater phenotypic variability than inbred strains [21]. An outbred stock was used so that the effects observed would not be specific to a single inbred genotype, as in our earlier work on sucrose solution intake in outbred rats [15], and Slc:ICR in particular because it is in routine use in this laboratory and is among the most widely used general-purpose outbred mouse stocks. All animals were naïve and had no prior experimental procedures. Mice were housed individually in stainless-steel wire-mesh cages at 22.5 ± 0.5 °C with a relative humidity of 55% ± 5% under a 12 h light/dark cycle (lights on 08:00–20:00). As environmental enrichment to promote animal welfare, a paper tube was provided in each cage and was replaced as needed. During the acclimation period, the mice were provided ad libitum access to standard rodent chow (CE-2; Japan Crea Inc., Tokyo, Japan) and tap water. All animal experiments were approved by the Animal Experiment Ethics Committee of Kobe Gakuin University (approval number: 26-02).

### 2.2. Experimental Design

After a 1-week acclimation period, the mice were randomly assigned, without stratification by body weight, to two groups: control (C, *n* = 24) and sucrose (S, *n* = 24). The initial body weight did not differ significantly between the groups . The investigators were not blinded to group allocation during data collection or analysis. During the experimental period, one mouse in group S showed signs of poor health, as determined by regular monitoring of body weight, food intake, and water intake, and was excluded from the analysis. The final number of mice in group S was 23. Mice in group C had free access to CE-2 and tap water, whereas mice in group S had free access to CE-2 and a 10% (w/v) sucrose solution (sucrose dissolved in tap water) for 27 days. The exposure window was selected based on the developmental trajectory of circulating testosterone in male mice, which in C57BL/6 and CD-1 begins to rise at about postnatal day 30, peaks at about day 40, and levels off at about day 60 [22]. The 27-day intervention, beginning at 4 weeks of age (approximately postnatal days 28 to 55), therefore starts immediately before that rise and spans both the rise and the peak. Body weight and food and water intakes were recorded periodically throughout the experimental period. On day 27, mice were fasted; at this time, the sucrose solution provided to group S was replaced with tap water. The mice were fasted for 16 h. This duration was chosen because it is the longest interval in a characterized series of fasts (0, 4, 8, 12 and 16 h) in male mice [23], placing all animals in the gluconeogenic and ketogenic phase rather than in the heterogeneous postprandial states of ad libitum-fed animals. On day 28, the mice were euthanized by exsanguination via cardiac blood collection under isoflurane anesthesia. All animals were euthanized between 08:30 and 12:30, i.e., 0.5–4.5 h after lights on, alternating between the two groups. Blood glucose and β-hydroxybutyrate (BHB) concentrations were measured immediately by using a handheld glucose or BHB monitoring device (FreeStyle Optium; Abbott, Tokyo, Japan). Subsequently, the liver, testes, epididymides, and epididymal fat were collected, and plasma was prepared from the cardiac blood. The weights of the liver, testes, epididymides, and epididymal fat were measured.

### 2.3. Plasma and Tissue Lipid Measurements

Plasma TG, total Chol, and high-density lipoprotein cholesterol (HDL-C) levels were measured using LabAssay kits (Fujifilm Wako, Tokyo, Japan), according to the manufacturer’s instructions. Blood samples showing visible hemolysis were excluded from the analyses of plasma TG, total Chol, and HDL-C concentrations because hemolysis can interfere with enzymatic measurements. Samples from 13 and 12 group C and group S mice, respectively, were used in these analyses. Hepatic and testicular lipid contents were determined using the same kits as those used for TG and Chol. Lipid extraction was performed prior to quantification. Liver tissue (100–130 mg) and testicular tissue (20–50 mg) were weighed and homogenized in 80 µL of distilled water. Methanol (Nacalai Tesque, Kyoto, Japan; 200 µL) and chloroform (Nacalai Tesque; 100 µL) were added, and the mixture was shaken at 1600 rpm for 30 min. Additional chloroform (100 µL) and distilled water (120 µL) were added, and the mixture was centrifuged (15,000 rpm, 5 min; Himac CF 15R; HITACHI, Ibaraki, Japan). The organic layer was collected and evaporated to dryness at 60 °C. The dried lipid residue was dissolved in 0.5 mL of 2-propanol (Nacalai Tesque) to prepare the sample for measurement. Extracts with a TG value identified as a statistical outlier (Tukey’s method, 1.5× interquartile range) were considered contaminated by peritesticular adipose tissue and excluded from the testicular Chol analysis; TG and Chol content were strongly correlated across all animals (Supplementary Figure S1), which supports this approach. Plasma lipid levels and tissue lipid content are expressed as mg/dL and per gram of wet tissue weight, respectively.

### 2.4. Plasma and Testicular Testosterone Measurements

The testosterone levels were measured using a Testosterone enzyme-linked immunosorbent assay (ELISA) Kit (#582701; Cayman Chemical, Ann Arbor, MI, USA). Prior to measurement, testosterone was extracted from testicular tissue and plasma by organic solvent extraction. Testicular tissue was weighed and homogenized in 1× phosphate-buffered saline (PBS; Nacalai Tesque) at five volumes (v/w) relative to the tissue weight. Plasma (100 μL) was mixed with an equal volume of LC-MS-grade water (Thermo Fisher Scientific, Waltham, MA, USA). Dehydrated diethyl ether (Nacalai Tesque, Kyoto, Japan) was then added to each testicular homogenate or plasma sample at a volume five times that of the sample, and the samples were vortexed and centrifuged (2000 rpm, 5 min, 4 °C; Himac CF 7D2; Hitachi, Ibaraki, Japan). The upper ether layer was collected, and the extraction was repeated twice. The combined ether layers were evaporated to dryness at 45 °C using a heating block, and the dried residue was reconstituted in 0.5 mL of 1× ELISA buffer (provided with the kit). The testicular samples were further diluted 50-fold prior to analysis. The ELISA was performed according to the manufacturer’s instructions. Plasma and testicular testosterone were each measured in a single assay run, using strip wells from a single kit lot, with all samples from both groups incubated and read together against a common standard curve. Each sample was assayed in a single well, so intra- and inter-assay coefficients of variation could not be determined in this laboratory; the manufacturer specifies an intra-assay coefficient of variation of 4.4–19.1% over the 3.9–500 pg/mL range of the assay. Samples whose absorbance fell outside the working range of the standard curve were excluded from analysis (Supplementary Table S1). The results are expressed as pg/mL for plasma testosterone and ng/g wet testis weight for intratesticular testosterone.

### 2.5. RNA Extraction, Reverse Transcription, and Gene Expression Analysis

Total RNA was extracted from the liver and testicular tissue using the Sepasol^®^ RNA I Super G reagent (Nacalai Tesque) according to the manufacturer’s instructions. An RNase inhibitor (SIN-201; TOYOBO, Osaka, Japan) was added to prevent RNA degradation. The concentration and purity of extracted RNA were determined using a NanoDrop spectrophotometer (Thermo Fisher Scientific). Reverse transcription and genomic DNA removal were performed using the ReverTra Ace^®^ qPCR RT Master Mix with gDNA Remover (TOYOBO) to generate cDNA. Gene expression analysis was performed using the StepOne Real-Time PCR System (Applied Biosystems, Foster City, CA, USA) and THUNDERBIRD™ Next SYBR^®^ qPCR Mix (TOYOBO). The primers used for qPCR analysis are listed in Table 1. Reference gene stability was assessed separately for each tissue before any target gene was analyzed, because the suitability of a reference gene is tissue-dependent. In the testes, *Actb* expression did not differ between groups and was used as the sole reference gene. In the liver, five candidate reference genes (*Actb*, *Hprt1*, *Rpl19*, *Ppia* and *Tbp*) were measured in every animal and their expression stability was compared by geNorm [24]. *Ppia* and *Rpl19* were the two most stable, and the pairwise variation indicated that two reference genes were sufficient; a normalization factor defined as the mean Cq of these two genes was therefore used. The complete details are provided in Supplementary Table S2 and Supplementary Methods. Relative mRNA expression was calculated by the ΔΔCq method. Amplification efficiency was determined for every reference gene in each tissue in which it was used (Supplementary Figure S2).

### 2.6. Western Blotting

Liver and testicular tissues were homogenized in radioimmunoprecipitation assay (RIPA) buffer containing protease inhibitors. Protein concentrations in the lysates were determined using a BCA Protein Assay Kit (Nacalai Tesque). Equal amounts of protein (30 μg) were separated using sodium dodecyl sulfate-polyacrylamide gel electrophoresis (SDS-PAGE) and transferred onto polyvinylidene difluoride (PVDF) membranes. The membranes were blocked with 2% polyvinylpyrrolidone in Tris-buffered saline containing 0.1% Tween-20 (TBST) for 1 h at room temperature and incubated overnight at 4 °C with primary antibodies against AMPK alpha (10929-2-AP, Proteintech, Rosemont, IL, USA), phospho-AMPK (Thr172; #2535, Cell Signaling Technology, Danvers, MA, USA), ACC (#3676, Cell Signaling Technology), phospho-ACC (#11818, Cell Signaling Technology), HSD3B (ab167417, Abcam, Cambridge, UK), CYP11A1 (13363-1-AP, Proteintech), CYP17A1 (14447-1-AP, Proteintech), and β-actin (010-27841, Fujifilm Wako). After washing with TBST, the membranes were incubated with horseradish peroxidase-conjugated anti-mouse IgG (115-035-003) or anti-rabbit IgG (111-035-003) secondary antibodies (Jackson ImmunoResearch Laboratories, West Grove, PA, USA) for 30 min at room temperature. Following three washes with TBST, chemiluminescent signals were detected using ImmunoStar LD or Zeta (Fujifilm Wako) according to the manufacturer’s instructions and visualized using a LumiCube Plus imaging system (Liponics Co., Ltd., Tokyo, Japan). Band intensities were quantified using ImageJ software (version 1.54g; National Institutes of Health, Bethesda, MD, USA). Total AMPK and ACC protein levels were normalized to β-actin, and the phosphorylation levels of AMPK and ACC were expressed as the ratios of phospho-AMPK to total AMPK and phospho-ACC to total ACC, respectively. The immunoreactive AMPKα signal at the expected molecular weight resolved into two closely spaced components, and the whole of that signal was enclosed in a single region of interest.

### 2.7. Statistical Analysis

All numerical data are presented as the mean ± standard error of the mean (SEM). Outliers were identified using Tukey’s method (1.5× interquartile range [IQR]) applied separately within each group; values falling outside [Q1 − 1.5 × IQR, Q3 + 1.5 × IQR] were excluded from the corresponding analysis, and the sample sizes for each variable are indicated in the figure legends. The exclusion of these animals did not alter the direction of any reported effect. For each two-group comparison, normality was assessed using the Shapiro–Wilk test applied separately to each group, and the homogeneity of variance was assessed using Levene’s test. When the Shapiro–Wilk test yielded *p* < 0.05 in either group, the Mann–Whitney U test was used. When normality was not rejected, Student’s *t*-test was used if Levene’s test yielded *p* > 0.05, and Welch’s *t*-test was used if Levene’s test yielded *p* < 0.05. All tests were two-tailed, and *p* < 0.05 was considered statistically significant; exact *p* values are reported in the figures. The genes examined were selected a priori as components of defined Chol-handling and steroidogenic pathways rather than screened without a prior hypothesis; therefore, no adjustment for multiple comparisons was applied. Given the number of endpoints assessed, isolated significant findings should nonetheless be interpreted with corresponding caution; the interpretation presented in the Discussion rests on the pattern of change across each panel and on the directly measured lipid and hormone concentrations rather than on individual transcript results. Statistical analyses were conducted using JASP (Version 0.95.1; JASP Team, 2025, University of Amsterdam, Amsterdam, The Netherlands).

## 3. Results

### 3.1. Effects of 10% Sucrose Solution Intake on Body Weight and Organ Weights

The effects of 10% sucrose solution on body weight, food intake, fluid intake, and organ weight in pubertal male mice are presented in Table 2. Over the 27-day intervention, mice in group S consumed 440 g of sucrose solution per animal (Table 2), corresponding to approximately 44.0 g of sucrose or 176 kcal, which accounted for approximately 25.0% of the total energy intake. The initial and final body weights (BW) and BW gain did not differ significantly between groups. Total food intake was significantly lower in group S than in group C, whereas total fluid intake was significantly higher in group S. However, the total energy intake and energy efficiency (BW gain per 100 kcal of energy intake) did not differ significantly between the groups. The liver, testis, and epididymis weights did not differ significantly between the groups. In contrast, peritesticular fat weight was significantly higher in group S than in group C. Blood glucose levels did not differ between the groups, whereas the blood BHB concentration was significantly higher in group S. Therefore, under the conditions of this study, sucrose intake increased peritesticular fat accumulation and blood BHB without affecting overall BW gain, energy intake, or the weights of the liver and reproductive organs.

**Table 2:** Body weight, intake parameters, and organ weights of control and sucrose-treated mice.

	Control Group	Sucrose Group	*p*-Value
Initial body weight (g)	23.3 ± 0.331	24.0 ± 0.344	0.113
Final body weight (g)	44.3 ± 0.739	43.9 ± 0.887	0.843
Body weight gain (g)	21.0 ± 0.661	20.4 ± 0.632	0.528
Total food intake (g/animal)	207 ± 2.97	156 ± 4.47	<0.001
Total water intake (g/animal)	261 ± 9.36	440 ± 31.5	<0.001
Total energy intake (kcal/animal)	714 ± 10.2	704 ± 10.8	0.528
Energy efficiency (g/100 kcal)	2.94 ± 0.086	2.90 ± 0.079	0.707
Liver weight (g/kg BW)	47.1 ± 0.782	47.8 ± 0.803	0.441
Testis weight (g/kg BW)	6.52 ± 0.198	6.38 ± 0.167	0.580
Epididymis weight (g/kg BW)	2.13 ± 0.044	2.14 ± 0.041	0.760
Peritesticular fat (g/kg BW)	19.3 ± 1.00	23.3 ± 1.29	0.0122
Blood glucose (mg/dL)	130 ± 6.92	127 ± 9.59	0.450
Blood BHB (mmol/L)	1.60 ± 0.102	2.23 ± 0.168	0.00430

### 3.2. Effects of 10% Sucrose Solution Intake on Plasma and Hepatic Lipid Profiles

**Figure 1:**
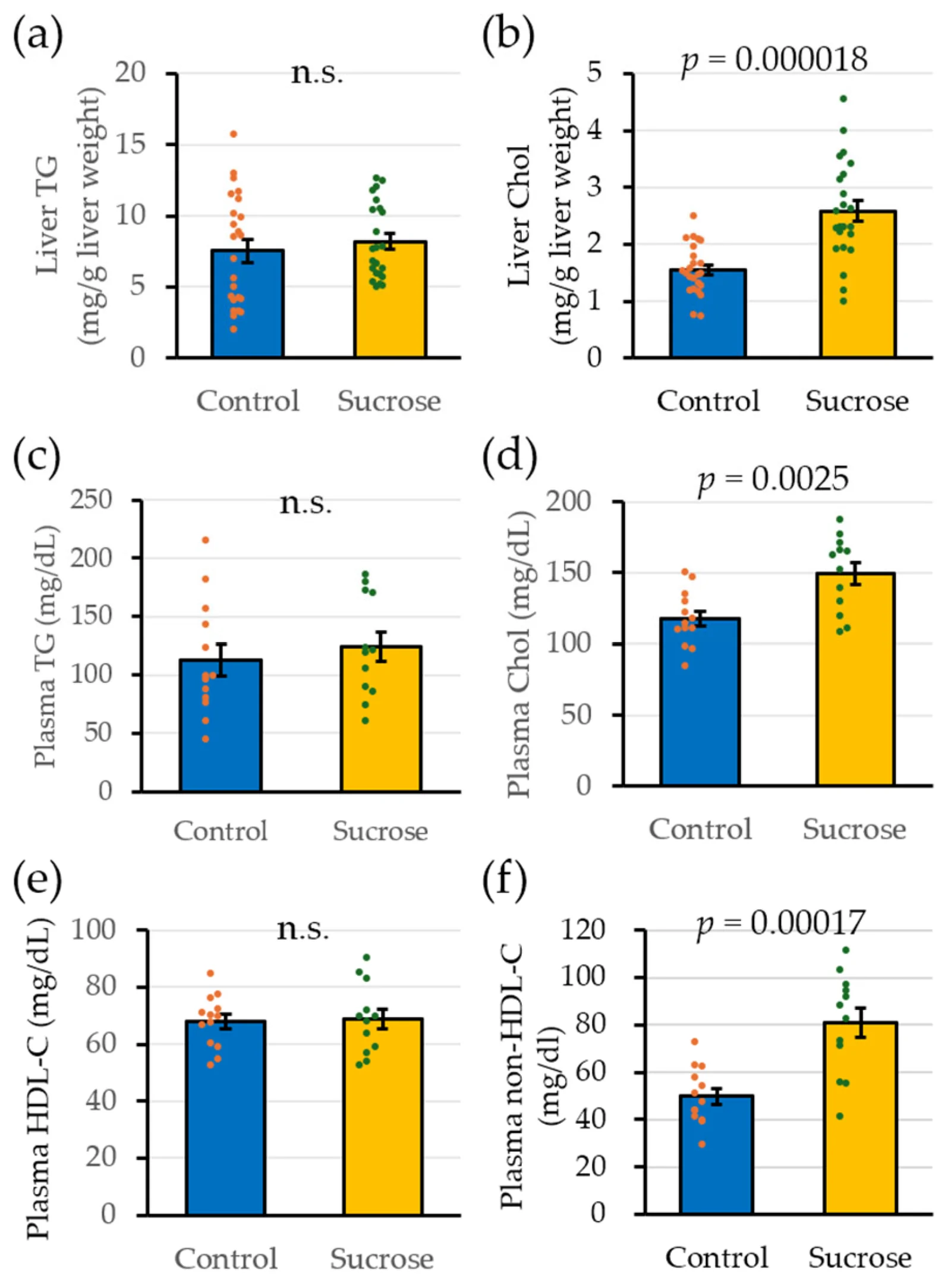
Effects of 10% sucrose solution intake on hepatic and plasma lipid profiles in pubertal male mice. (**a**) Hepatic triglyceride (TG) content, (**b**) hepatic cholesterol (Chol) content, (**c**) plasma TG concentration, (**d**) plasma total Chol concentration, (**e**) plasma HDL-cholesterol (HDL-C) concentration, and (**f**) plasma non-HDL-cholesterol (non-HDL-C) concentration, calculated as total Chol minus HDL-C. Data are presented as mean ± SEM with individual data points overlaid (Control, orange; Sucrose, green). Exact *p*-values are shown above each panel; n.s., not significant. Hepatic TG, Mann–Whitney *U* test (Control n = 24, Sucrose n = 23); hepatic Chol, Welch’s *t*-test (Control n = 24, Sucrose n = 23); plasma TG, Student’s *t*-test (Control n = 13, Sucrose n = 12); plasma total Chol, Student’s *t*-test (Control n = 13, Sucrose n = 12); plasma HDL-C, Student’s *t*-test (Control n = 13, Sucrose n = 12); plasma non-HDL-C, Student’s *t*-test (Control n = 13, Sucrose n = 12).

Sucrose or fructose intake has been reported to alter hepatic lipid metabolism, promoting increased DNL and elevated circulating Chol levels in both animal models and human studies [25,26]. Therefore, we examined the effects of 10% sucrose solution intake on hepatic and plasma lipid profiles in pubertal male mice (Figure 1). Hepatic Chol content was significantly higher in group S than in group C (Figure 1b), whereas hepatic TG content showed no significant difference between the groups (Figure 1a). Although plasma total Chol and non-HDL-cholesterol (non-HDL-C) concentrations were significantly elevated in group S (Figure 1d,f), there were no significant between-group differences in plasma HDL-C and TG concentrations (Figure 1c,e). These results suggest that the intake of 10% sucrose solution selectively increased Chol levels in both the liver and plasma of pubertal male mice, with the increase in circulating Chol confined to the non-HDL fraction.

### 3.3. Effects of 10% Sucrose Solution Intake on Hepatic Chol Metabolism-Related Genes

**Figure 2:**
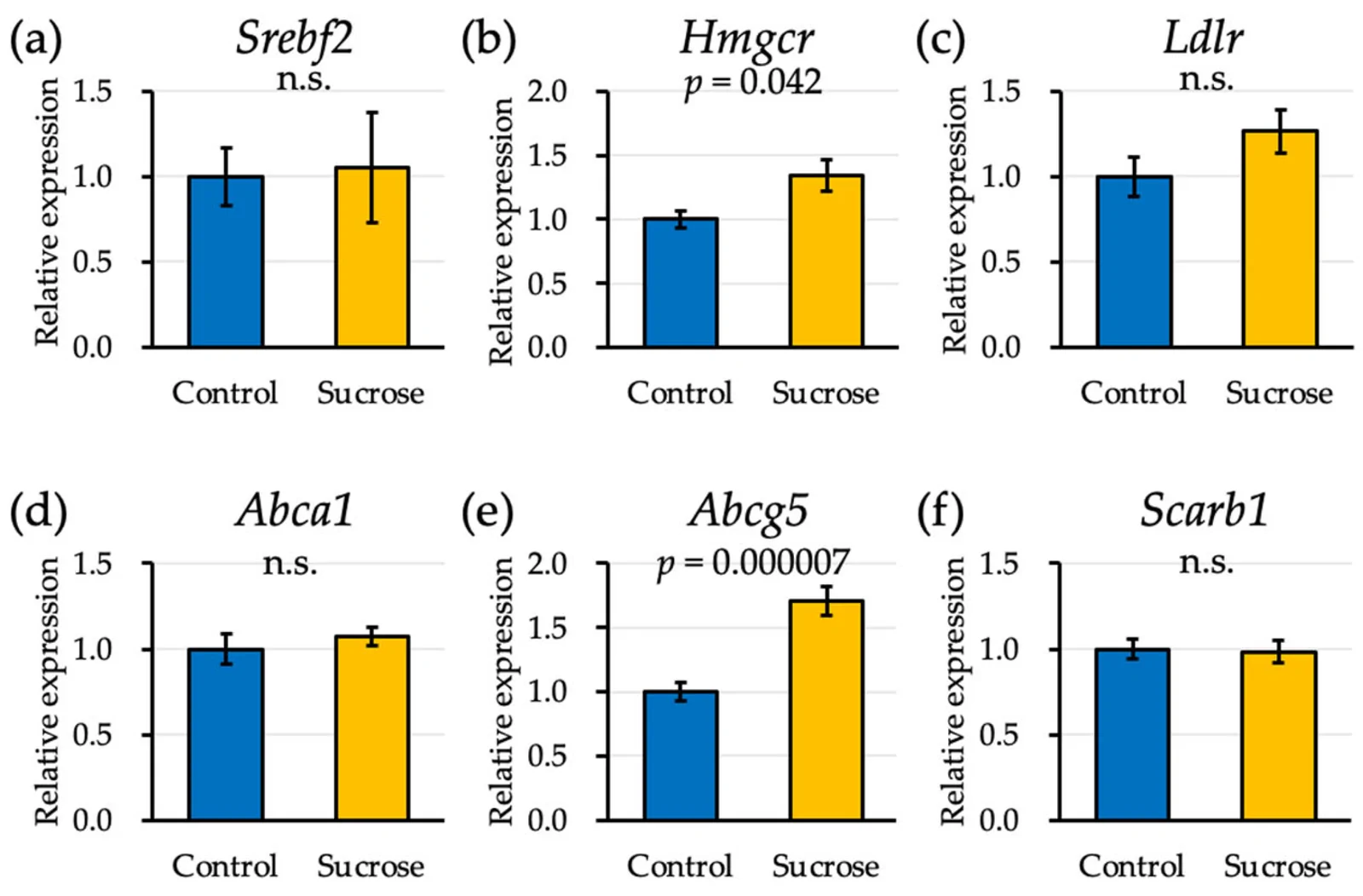
Effects of 10% sucrose solution intake on hepatic Chol metabolism-related gene expression in pubertal male mice. Relative mRNA expression levels of *Srebf2* (**a**), *Hmgcr* (**b**), *Ldlr* (**c**), *Abca1* (**d**), *Abcg5* (**e**), and *Scarb1* (**f**) in Control and Sucrose-fed mice, normalized to a two-gene normalization factor derived from *Ppia* and *Rpl19* and expressed relative to Control (=1). Data presented are mean ± SEM with exact *p*-values shown above each panel. Sample sizes and the statistical test applied to each panel are indicated below; n.s., not significant. *Srebf2*, Mann–Whitney U test (Control n = 24, Sucrose n = 23); *Hmgcr*, Mann–Whitney U test (Control n = 24, Sucrose n = 23); *Ldlr*, Student’s *t*-test (Control n = 21, Sucrose n = 22); *Abca1*, Mann–Whitney U test (Control n = 22, Sucrose n = 22); *Abcg5*, Welch’s *t*-test (Control n = 21, Sucrose n = 23); *Scarb1*, Student’s *t*-test (Control n = 23, Sucrose n = 23). Statistical outliers were identified and excluded for each gene using Tukey’s method (1.5× interquartile range), as described in the Methods section.

Intake of the 10% sucrose solution selectively increased hepatic and plasma Chol levels (Figure 1). Hepatic Chol homeostasis is maintained by a balance between supply (through synthesis and uptake) and removal (through HDL formation and biliary excretion). To clarify the hepatic Chol metabolic dynamics underlying this increase, we analyzed the expression of the genes involved in these processes (*Srebf2*, *Hmgcr*, *Ldlr*, *Abca1*, *Abcg5*, and *Scarb1*; Figure 2). *Srebf2*, *Ldlr*, *Abca1* and *Scarb1* mRNA levels did not differ between groups (Figure 2a,c,d,f). *Hmgcr* mRNA levels were modestly higher in group S (approximately 1.3-fold; Figure 2b), and *Abcg5* mRNA levels showed the largest increase (approximately 1.7-fold; Figure 2e), the only transcript in this panel whose difference is large relative to the between-animal variation. These changes were selective rather than uniform: of the six transcripts that were examined, four were unchanged, and the largest change was in *Abcg5*, which encodes a component of the biliary sterol export machinery. Because the proteins encoded by these genes are subject to substantial posttranscriptional and posttranslational regulation, these data indicate a transcriptional response to sucrose intake but do not by themselves establish corresponding changes in Chol synthesis, uptake, or excretion.

### 3.4. Effects of 10% Sucrose Solution Intake on Hepatic TG Metabolism

**Figure 3:**
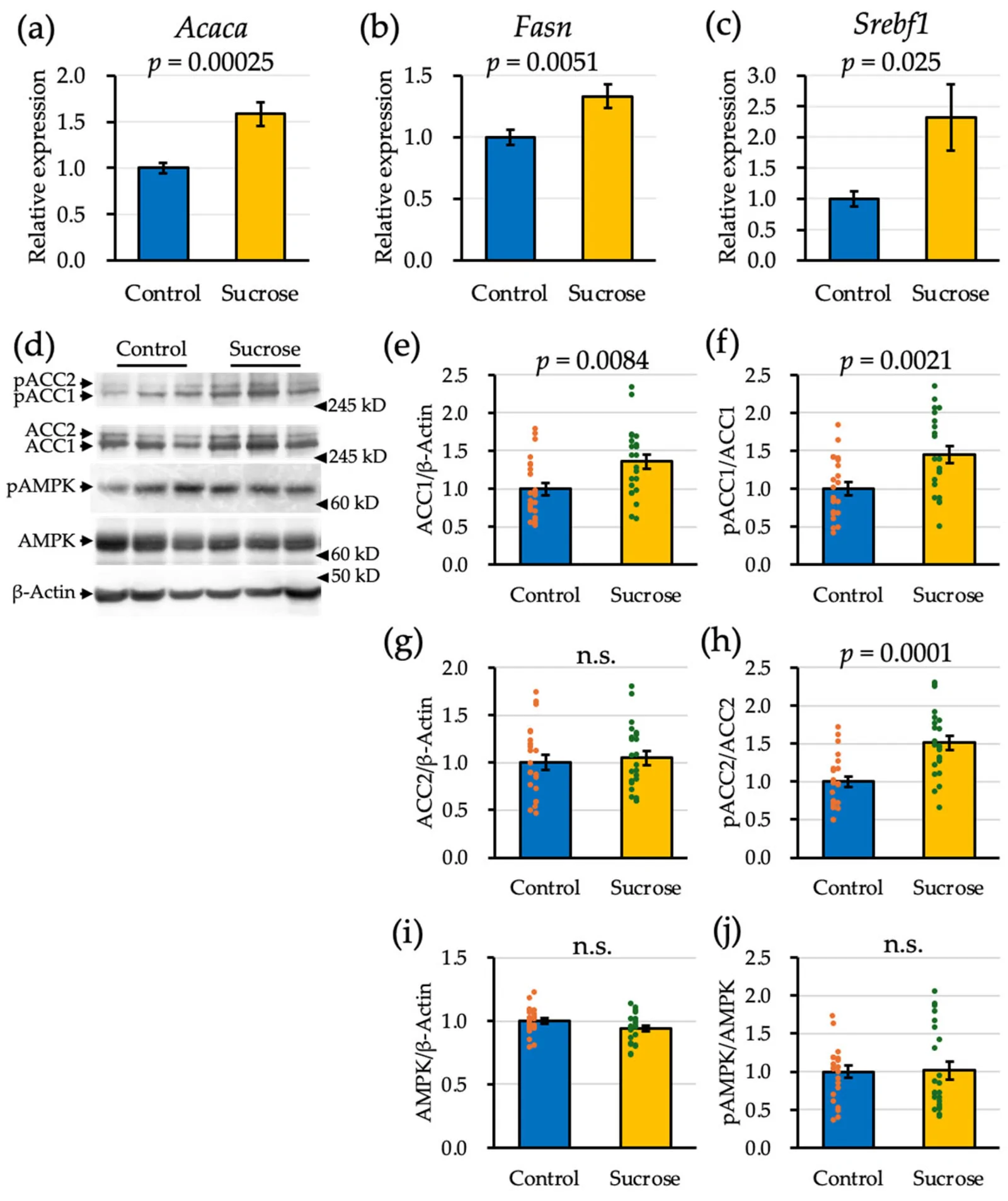
Effects of sucrose intake on hepatic lipogenic gene expression and ACC/AMPK phosphorylation in a pubertal mouse model. (**a**–**c**) Effects of sucrose intake on mRNA expression of *Acaca*, *Fasn*, and *Srebf1* in Control and Sucrose-fed mice, normalized to a two-gene normalization factor derived from *Ppia* and *Rpl19* and expressed relative to Control (=1). *Srebf1* was measured with primers specific to the transcript variant encoding SREBP-1c. (**d**) Representative Western blot images for pACC2, pACC1, ACC2, ACC1, pAMPK, AMPK, and β-actin from Control and Sucrose liver lysates. (**e**–**j**) Densitometric quantification of (**e**) ACC1/β-actin, (**f**) pACC1/ACC1, (**g**) ACC2/β-actin, (**h**) pACC2/ACC2, (**i**) AMPK/β-actin, and (**j**) pAMPK/AMPK, expressed relative to Control. Data shown are mean ± SEM with individual data points overlaid (Control, orange; Sucrose, green), with the exact *p*-value shown above each panel. Sample sizes and the statistical test applied to each panel are indicated below; n.s., not significant. *Acaca*, Welch’s *t*-test (Control n = 23, Sucrose n = 22); *Fasn*, Student’s *t*-test (Control n = 22, Sucrose n = 21); *Srebf1*, Mann–Whitney U test (Control n = 22, Sucrose n = 23); ACC1/β-actin, Mann–Whitney U test (Control n = 23, Sucrose n = 23); pACC1/ACC1, Student’s *t*-test (Control n = 23, Sucrose n = 22); ACC2/β-actin, Student’s *t*-test (Control n = 23, Sucrose n = 23); pACC2/ACC2, Student’s *t*-test (Control n = 24, Sucrose n = 23); AMPKα/β-actin, Student’s *t*-test (Control n = 22, Sucrose n = 23); pAMPKα/AMPKα, Mann–Whitney U test (Control n = 22, Sucrose n = 23). Statistical outliers were identified and excluded for each panel using Tukey’s method (1.5× interquartile range), as described in the Methods section.

To assess whether hepatic DNL was activated, we examined the expression of *Acaca* and *Fasn*, which encode rate-limiting enzymes involved in fatty acid synthesis. Both genes showed a significant increase in group S compared with group C (Figure 3a,b). We further examined the expression of *Srebf1*, measured with primers specific to the transcript variant encoding sterol regulatory element-binding protein 1c (SREBP-1c), a key transcriptional regulator of hepatic lipogenic gene expression. *Srebf1* expression was significantly higher in group S than in group C (Figure 3c). Next, we examined the protein expression and phosphorylation status of acetyl-CoA carboxylase 1 (ACC1), the rate-limiting enzyme in fatty acid synthesis. ACC1 protein levels were significantly higher in group S than in group C (Figure 3d,e). However, the phosphorylation level of ACC1, which reflects its inhibition by phosphorylation, significantly increased in group S (Figure 3f). In contrast, the protein expression of ACC2, which regulates mitochondrial fatty acid oxidation through the production of malonyl-CoA and the subsequent control of CPT1-mediated fatty acid entry into the mitochondria, was unchanged between the groups (Figure 3g), whereas the phosphorylation level of ACC2 was significantly increased in group S (Figure 3h). Because AMPK is a major upstream kinase responsible for ACC phosphorylation [27], we next examined AMPKα. AMPKα protein expression did not differ significantly between the groups (*p* = 0.069; Figure 3i), nor did the phosphorylation level of AMPKα at Thr172, a marker of AMPK activation (Figure 3j). Collectively, these findings suggest that the intake of 10% sucrose solution alters hepatic fatty acid metabolism at multiple levels. Although the expression of lipogenic genes and ACC1 protein increased, the concomitant increase in ACC1 phosphorylation suggests that ACC1 activity may be subject to inhibitory regulation. Increased ACC2 phosphorylation would be expected to lower malonyl-CoA at the mitochondrial outer membrane and thereby relieve CPT1 inhibition; correspondingly, the plasma β-hydroxybutyrate concentration was significantly higher in group S than in group C (Table 2). Neither hepatic nor plasma TG concentrations were altered (Figure 1) despite transcriptional activation of the lipogenic program. Notably, these alterations were not accompanied by increased AMPKα Thr172 phosphorylation, which suggests that the observed changes in ACC phosphorylation may have occurred via AMPK-independent mechanisms.

### 3.5. Effects of 10% Sucrose Solution Intake on Testicular Expression of Chol Metabolism-Related Genes

**Figure 4:**
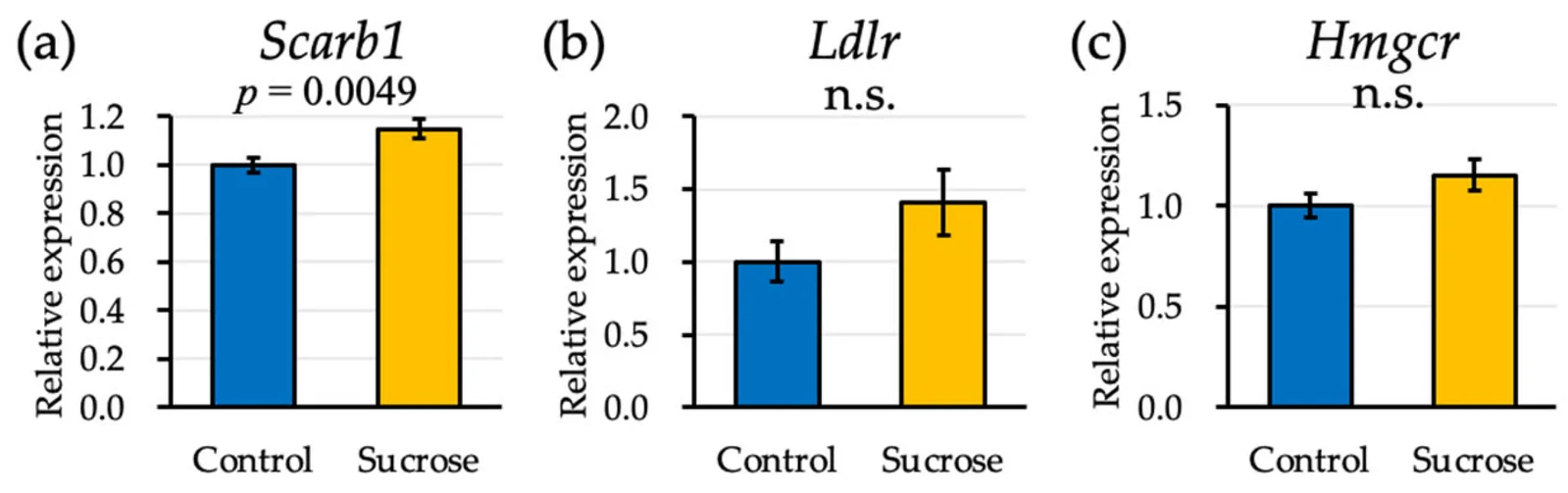
Effects of 10% sucrose solution intake on the mRNA expression of Chol metabolism-related genes in testicular tissue. Relative mRNA expression levels of *Scarb1* (**a**), *Ldlr* (**b**), and *Hmgcr* (**c**) in Control and Sucrose-fed mice, normalized to *Actb* and expressed relative to Control (=1). Data presented are mean ± SEM with exact *p*-values shown above each panel; n.s., not significant. *Scarb1*, Student’s *t*-test (Control n = 23, Sucrose n = 23); *Ldlr*, Mann–Whitney *U* test (Control n = 21, Sucrose n = 23); *Hmgcr*, Mann–Whitney U test (Control n = 23, Sucrose n = 21). Statistical outliers were identified and excluded for each gene using Tukey’s method (1.5× interquartile range), as described in the Methods section.

Because the intake of sucrose solution increased hepatic Chol content and circulating non-HDL-C concentrations (Figure 1d,f), we hypothesized that Chol availability to testicular Leydig cells might be altered. Leydig cells acquire Chol from both low-density lipoprotein (LDL) and HDL in the circulation. LDL is taken up via the LDL receptor (LDLR), encoded by *Ldlr*, whereas HDL-derived Chol esters are acquired by selective uptake through SR-BI, encoded by *Scarb1*, which is the principal route supporting steroidogenesis in rodents [12,28,29,30,31]. Therefore, the mRNA expression of Chol metabolism-related genes in the testicular tissue was analyzed (Figure 4). The expression of *Scarb1* was modestly but significantly increased in group S compared with group C (Figure 4a), whereas the expression of the LDL receptor gene, *Ldlr*, and the Chol biosynthetic enzyme gene, *Hmgcr*, did not differ between the groups (Figure 4b,c). These findings indicate a modest increase in *Scarb1* transcript abundance, of the order of 15%, with no detectable change in *Ldlr* or *Hmgcr*. A transcript difference of this size should not be read as evidence of a corresponding change in SR-BI-mediated Chol uptake, which is governed largely at the protein level.

### 3.6. Effects of 10% Sucrose Solution Intake on Testicular Steroidogenic Enzyme Gene Expression

**Figure 5:**
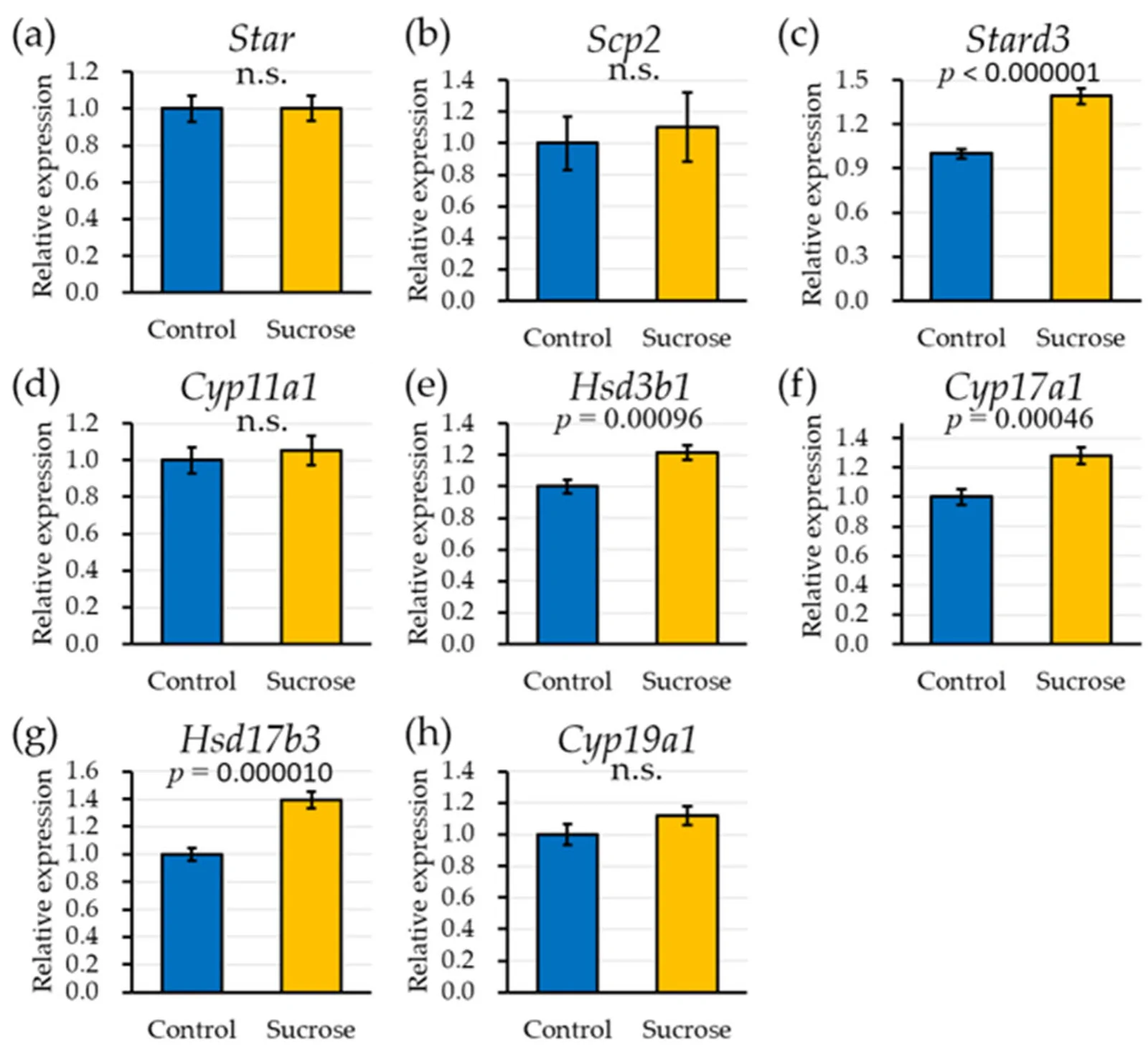
Effects of 10% sucrose solution intake on testicular steroidogenic enzyme gene expression in pubertal male mice. Relative mRNA expression levels of *Star* (**a**), *Scp2* (**b**), *Stard3* (**c**), *Cyp11a1* (**d**), *Hsd3b1* (**e**), *Cyp17a1* (**f**), *Hsd17b3* (**g**), and *Cyp19a1* (**h**) in Control and Sucrose-fed mice, normalized to *Actb* and expressed relative to Control (=1). Data presented are mean ± SEM with exact *p*-values shown above each panel; n.s., not significant. *Star*, Mann–Whitney U test (Control n = 24, Sucrose n = 22); *Scp2*, Mann–Whitney U test (Control n = 22, Sucrose n = 21); *Stard3*, Welch’s *t*-test (Control n = 20, Sucrose n = 23); *Cyp11a1*, Student’s *t*-test (Control n = 24, Sucrose n = 23); *Hsd3b1*, Student’s *t*-test (Control n = 23, Sucrose n = 23); *Cyp17a1*, Student’s *t*-test (Control n = 24, Sucrose n = 23); *Hsd17b3*, Student’s *t*-test (Control n = 22, Sucrose n = 23); *Cyp19a1*, Student’s *t*-test (Control n = 21, Sucrose n = 22). Statistical outliers were identified and excluded for each gene using Tukey’s method (1.5× interquartile range), as described in the Methods section.

To investigate whether the changes in systemic Chol metabolism that were induced by sucrose intake were accompanied by alterations in testicular Chol utilization and steroidogenesis, we examined the expression of genes involved in intracellular Chol trafficking and steroid hormone biosynthesis (Figure 5). Among the intracellular cholesterol trafficking-related genes examined, the expression of *Star* and *Scp2* did not differ between the groups, whereas *Stard3* expression significantly increased in group S (Figure 5a–c). The expression of *Cyp11a1*, which encodes an enzyme that catalyzes the first step of steroidogenesis, did not differ between the groups (Figure 5d). In contrast, the expression of genes encoding downstream steroidogenic enzymes, including *Hsd3b1*, *Cyp17a1*, and *Hsd17b3*, significantly increased in group S (Figure 5e–g). *Cyp19a1*, which encodes an aromatase that converts testosterone to 17β-estradiol, showed no significant change (Figure 5h). Although the mRNA expression of several downstream steroidogenic enzymes increased, the protein expression levels of CYP11A1, CYP17A1, and HSD3B did not differ significantly between groups (Supplementary Figure S3). The expression of *Lhcgr*, which encodes the luteinizing hormone (LH) receptor that serves as the primary upstream regulator of Leydig cell steroidogenesis, did not significantly differ between the groups (group C, 1.00 ± 0.0570; group S, 1.09 ± 0.0673 relative expression; *p* = 0.304). These findings indicate that sucrose intake was associated with selective changes in testicular Chol trafficking and the transcription of downstream steroidogenic enzymes, without corresponding changes in *Cyp11a1* or *Cyp19a1* transcript levels or in the protein levels of the enzymes examined.

### 3.7. Effects of 10% Sucrose Solution Intake on Testicular Chol Content and Testicular and Plasma Testosterone Concentrations

**Figure 6:**
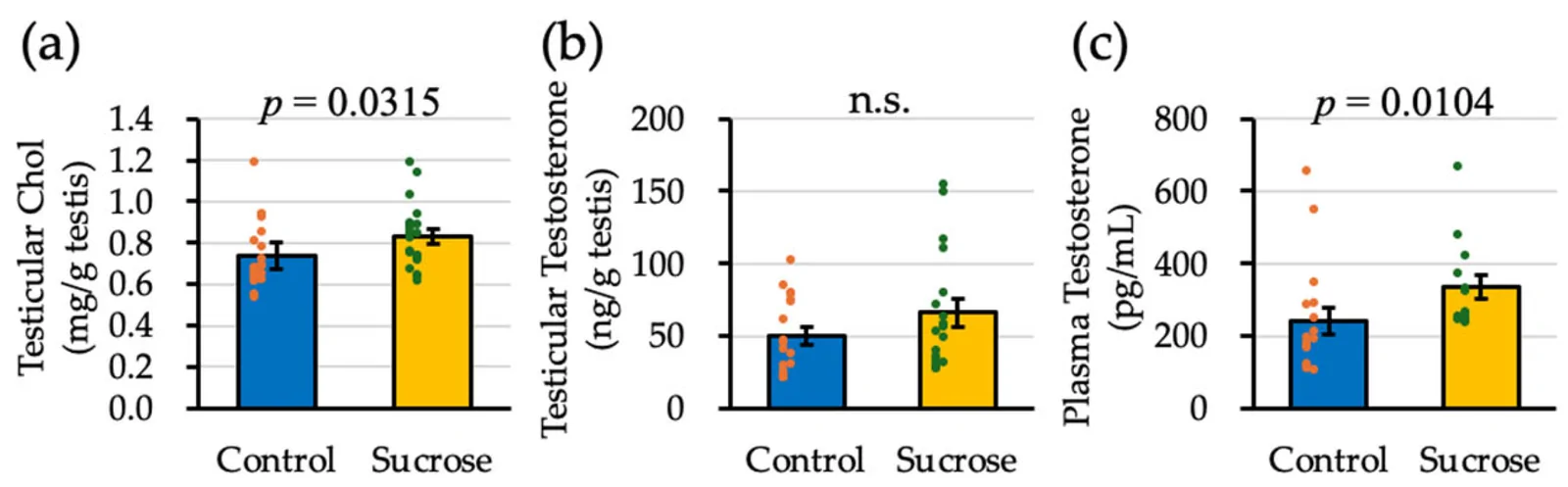
Effects of 10% sucrose solution intake on testicular cholesterol content and testosterone concentrations in pubertal male mice. (**a**) Testicular cholesterol content, (**b**) testicular testosterone concentration, and (**c**) plasma testosterone concentration. Data presented are mean ± SEM with individual data points overlaid (Control, orange; Sucrose, green) and exact *p*-values shown above each panel; n.s., not significant. Testicular cholesterol, Mann–Whitney U test (Control n = 21, Sucrose n = 22); testicular testosterone, Mann–Whitney U test (Control n = 19, Sucrose n = 18); plasma testosterone, Mann–Whitney U test (Control n = 17, Sucrose n = 14). Samples with a triglyceride (TG) content in the lipid extract identified as a statistical outlier (Tukey’s method, 1.5× interquartile range), indicative of contamination by peritesticular adipose tissue, were excluded from the testicular cholesterol measurement; for the testosterone measurements, samples whose %B/B_0_ fell outside the working range of the standard curve, so that no concentration could be interpolated, and samples whose interpolated value was a statistical outlier within its group (Tukey’s method), were both excluded; no plasma sample was available for one sucrose-group animal. The numbers excluded for each reason are given in Supplementary Table S1.

Because sucrose solution intake upregulated several testicular steroidogenic enzyme genes (Figure 5), testicular Chol content and testosterone concentrations were examined (Figure 6). The testicular Chol content was higher in group S than in group C (Figure 6a), although this difference depends on the exclusion of extracts identified as contaminated with peritesticular adipose tissue and is not significant when all extracts are retained (Supplementary Table S4). The testicular testosterone content was not significantly different between the groups (Figure 6b), whereas the plasma testosterone concentration was significantly higher in group S than in group C (Figure 6c). These results indicate that 10% sucrose solution intake during puberty is associated with a higher circulating testosterone concentration, without a corresponding increase in intratesticular testosterone.

## 4. Discussion

This study demonstrated that free access to a 10% (w/v) sucrose solution during puberty—one-third the concentration used in the two most closely comparable studies [16,19], but supplying approximately 25% of total energy intake—elevated hepatic and plasma Chol levels without affecting TG levels and increased plasma testosterone concentrations in male ICR mice. The increase in circulating Chol was confined to the non-HDL fraction, whereas HDL-C levels remained unchanged. This Chol-predominant rather than TG-predominant lipid response, together with the concurrent increase in testicular Chol content and plasma testosterone concentration, is consistent with our hypothesis that pubertal sucrose exposure increases rather than suppresses circulating androgen levels. All measurements reported here were made after a 16 h fast during which the sucrose solution had been replaced with water, and therefore reflect the fasted state after sucrose withdrawal rather than the state during ongoing sucrose consumption.

Before considering the mechanisms underlying these changes, it should be noted that the 10% solution delivered a substantial fructose load. On hydrolysis, 10% (w/v) sucrose yields 5.3% (w/v) fructose and 5.3% (w/v) glucose, so the 44.0 g of sucrose consumed per animal over the 27-day intervention corresponds to approximately 23 g of fructose, or 0.86 g per day. Mice in group S also consumed approximately 1.7 times as much fluid as controls (Table 2); hepatic fructose exposure was therefore considerably greater than the solution concentration alone would suggest. Moreover, the amount delivered per drinking bout may have exceeded the saturable capacity for intestinal fructose metabolism [32].

The fasted state in which the hepatic findings were obtained bears on their interpretation. A 16 h fast moves the mouse liver into the gluconeogenic and ketogenic phase, and β-hydroxybutyrate, ACC phosphorylation and AMPKα are all responsive to it [23]; the group difference in these endpoints is therefore as readily a difference in the response to fasting as a difference in a steady state, and we do not attempt to distinguish the two. With that stated, a notable feature of the present model is that the hepatic lipogenic program was transcriptionally activated without the corresponding accumulation of TG. *Srebf1, Acaca* and *Fasn* were all significantly upregulated and ACC1 protein abundance increased, yet neither hepatic nor plasma TG concentrations differed between the groups. The phosphorylation status of ACC offers one explanation, since both the pACC1/ACC1 and pACC2/ACC2 ratios were significantly elevated in the S group. Because phosphorylation inactivates ACC [33], this would be expected to limit malonyl-CoA production, despite the increase in total ACC abundance; this is an inference from the phosphorylation ratio and is not tested here, since hepatic malonyl-CoA was not measured. Acetyl-CoA that is not utilized for fatty acid synthesis may then become available for other pathways because acetyl-CoA is the common precursor for both fatty acid and isoprenoid (including Chol) biosynthesis [34] and its partitioning varies with the metabolic state [35]. Consistent with this, *Hmgcr* expression and hepatic Chol content both increased, a pattern also reported in fructose-treated hepatocytes [36], although the effects of fructose on hepatic Chol synthesis were not consistent across studies [37]. Elevated pACC2 levels would likewise be expected to relieve CPT1 inhibition [33]; plasma BHB levels were higher in the S group (Table 2), which is consistent with that expectation but does not establish it, since fatty acid oxidation was not measured. In the S group neither the total amount of AMPKα protein nor the pAMPKα (Thr172)/AMPKα ratio differed between the groups, which indicates that the increased ACC phosphorylation is not due to enhanced AMPK activation.

This interpretation remains tentative. Total ACC1 protein levels were elevated; therefore, the absolute amount of catalytically active ACC1 could not be inferred from the phosphorylation ratio alone, and neither hepatic malonyl-CoA nor metabolic flux was measured. An alternative and not mutually exclusive explanation is that food intake was approximately 25% lower in the S group (Table 2) and the changes in ACC phosphorylation and plasma BHB levels were also characteristic of a reduced feeding state. Whether the observed changes reflected fructose-specific signaling or altered feeding patterns could not be resolved using the present design.

The transcriptional response of the hepatic Chol metabolism-related genes was selective rather than uniform, and two findings are particularly relevant to its interpretation. The first is *Abcg5*, which showed the largest increase in any transcript in this panel. The greatest change occurring in a component of the biliary sterol export machinery is more readily interpreted as a compensatory response than as a causal factor: *Abcg5* is regulated by the liver X receptor and mediates the excretion of neutral sterols into bile [38], so hepatic Chol accumulation would be expected to induce its expression. On this reading the induction of *Abcg5* is a consequence of the expanded hepatic Chol pool rather than its cause, and is consistent with the directly measured increase in hepatic Chol content. The second is *Srebf2*, whose transcript level remained unchanged although its downstream target *Hmgcr* was significantly upregulated. This was not unexpected, since SREBP-2 activity is governed by the proteolytic processing of the precursor and nuclear translocation of the mature form, rather than by transcript abundance [39].

Irrespective of the transcriptional mechanisms operating in the liver, the net result was an expansion of the circulating Chol pool. We next asked whether this was reflected in the testis. Testicular Chol content was higher in the S group, paralleling the expansion of the circulating Chol pool, although that difference is analysis-dependent (Supplementary Table S4). Among the testicular Chol-handling genes examined, only *Scarb1* was modestly upregulated, while *Ldlr* and *Hmgcr* remained unchanged. The route by which Chol reached the testis cannot be specified from these data: plasma HDL-C, the ligand for SR-BI [28], was unchanged while non-HDL-C increased; conversely, *Ldlr* expression did not increase despite the greater availability of apoB-containing lipoproteins. Because uptake depends on ligand concentration and receptor abundance, an increase in circulating Chol could enhance delivery without a corresponding change in receptor expression. We therefore interpret these findings as concordant observations—an expanded circulating Chol pool accompanied by increased testicular Chol—rather than as evidence of a specific uptake pathway.

Within the steroidogenic pathway, the transcriptional response was likewise selective. Among the trafficking-related genes, *Stard3* was increased whereas *Star* and *Scp2* remained unchanged. STARD3, also known as MLN64, is a late endosomal sterol-binding protein that creates endoplasmic reticulum–endosome membrane contact sites and delivers cholesterol to endosomes at the expense of the plasma membrane [40], and that mediates the egress of endosomal cholesterol to mitochondria [41]; its overexpression raises mitochondrial cholesterol content [42]. StAR, by contrast, governs the acute, rate-limiting delivery of Chol to the inner mitochondrial membrane, and is regulated acutely and post-transcriptionally rather than through transcript abundance [43]. We do not draw a trafficking inference from this pattern: STARD3 and StAR act at different steps and are regulated differently, and transcript measurements in whole-testis lysate, which cannot resolve the Leydig cell compartment, do not bear on whether the rate-limiting step was engaged. Among the enzymes, *Hsd3b1*, *Cyp17a1* and *Hsd17b3* were significantly increased whereas *Cyp11a1* and *Cyp19a1* remained unchanged; CYP11A1, CYP17A1 and HSD3B protein levels, however, did not differ between groups (Supplementary Figure S3), a dissociation between transcript and protein that parallels the *Srebf2*/SREBP-2 situation described above. The absence of any increase in steroidogenic enzyme protein, together with the concurrent increase in testicular Chol content, is more appropriately interpreted as an association between greater Chol availability and higher testosterone concentrations rather than as evidence of increased steroidogenic capacity.

Plasma testosterone was higher in group S, whereas intratesticular testosterone was not; the basis for this discrepancy remains unclear. Local homeostatic regulation of the intratesticular pool is one possibility, but this remains speculative: we measured neither LH nor testosterone production directly, and a higher circulating concentration can arise from altered clearance, binding or secretion as readily as from increased synthesis. What the data establish is an association between an expanded circulating Chol pool, a higher testicular Chol content and a higher plasma testosterone concentration, measured in the same animals; they do not establish a flux relationship between them. Therefore, enhanced testicular production could not be demonstrated directly, which remains an important limitation.

Nonetheless, the direction of the change in circulating testosterone is consistent with the hypothesis set out in the Introduction. Rodent studies confining sugar exposure to the juvenile–peripubertal window have reported elevated testosterone [16,17], whereas those extending exposure to adulthood have reported suppression [18,19]. The present findings fall on the former side of this divide, and the comparison can be drawn more sharply than we drew it. All four of these studies were carried out in rats, and they differ among themselves in sugar species, route and concentration, and in the ages at which exposure began and at which the animals were assessed (Supplementary Table S3). Two of them are matched in sugar, route and concentration: both gave a 30% (w/v) sucrose solution as the drinking fluid. De León-Ramírez et al. gave it to Wistar rats from weaning and assessed them at 51 days of age, and serum testosterone was elevated [16]; Adekunbi et al. gave the same solution to Sprague–Dawley rats from 8 weeks of age for 6 weeks, and testosterone was reduced [19]. Because these two studies differ chiefly in when the exposure took place, their divergence is difficult to attribute to the sugar or to the dose. The remaining two follow the same ordering: a 20% (w/v) fructose solution given from postnatal day 30 and assessed at day 60 elevated testosterone [17], whereas a 65% high-fructose solid diet given from 6 weeks of age for 21 weeks reduced it [18]. Across these four studies, the direction of the effect appears to be related more closely to the developmental stage at which the animals were assessed than to the type, concentration, or route of sugar exposure, and it does so in two different outbred rat stocks. The present study differs from all four in species, and the two species are not on the same timetable. In the male rat, puberty occupies approximately P42 to P55 and plasma testosterone rises between P40 and P60, stabilizing at P90; in the male mouse, plasma testosterone begins to rise at about P30, peaks at about P40 and levels off at about P60, the latter figures having been obtained in C57BL/6 and CD-1 mice, the latter stock being of ICR origin [22]. The exposure used here, from 4 to 8 weeks of age (P28 to P55), therefore begins immediately before the androgen trajectory of the male mouse starts to rise, spans its rise and its peak, and ends shortly before it levels off, so that the animals were assessed before the attainment of adulthood. In this context, the present study is more comparable to [16,17], in which the animals were assessed while the androgen trajectory was still incomplete, than to [18,19], in which assessment was performed after testosterone levels had stabilized. We note that the exposure was not confined to puberty in the strict sense: it spanned the pubertal transition and the adolescent period that follows it. Whether the ordering seen among the rat studies extends to the mouse cannot be settled by a single experiment, and the concordance reported here should be read as consistent with that possibility rather than as confirming it. As to the amount of sugar, the solution used here was one-third as concentrated as those in [16,19]; on a fructose-equivalent basis it delivered approximately one-quarter of the fructose concentration used in [17]. No comparison of concentration is meaningful with [18], in which the sugar was incorporated into the solid diet at 65%. During puberty, Leydig cells actively differentiate and expand their steroidogenic capacity [14], so an increase in substrate availability may be more readily translated into output than in adults, where the metabolic sequelae of chronic sugar intake could offset any such benefit. Given that adolescents are among the highest consumers of SSB [2,3], habitual intake during this period warrants further investigation.

This study had several limitations. First, the design does not isolate the effect of sucrose. Mice given the sucrose solution reduced their intake of solid chow by approximately 25%, so that total energy intake was unchanged but the intake of protein, fat, micronutrients and fiber from the solid diet was lower; an isocaloric and isonutrient control group, or at least an isocaloric glucose solution, would be required to separate the two. Thus, the present findings reflect sucrose consumption together with the accompanying reduction in chow intake, rather than the effect of sucrose alone. Second, causality was not established: the concordance between the expanded circulating Chol pool, the higher testicular Chol content and the elevated plasma testosterone level is compatible with a substrate-driven mechanism, but the testicular Chol difference is small and analysis-dependent (Supplementary Table S4), Chol flux was not traced, and no intervention was performed. Third, most endpoints were assessed at the transcript level, and the dissociations between transcript and protein observed here caution against inferring functional change from expression data alone; SR-BI protein, enzyme activity, hepatic malonyl-CoA, and SREBP-2 protein were not measured, so the proposed role of ACC phosphorylation remains provisional. Fourth, other components of the hypothalamic–pituitary–gonadal axis, including LH, FSH, and sex hormone-binding globulin, were not assessed, and no measure of reproductive function was obtained; whether the endocrine changes observed here have functional consequences for fertility is therefore unknown. Fifth, insulin and indices of insulin resistance, oxidative stress, and inflammatory markers were not measured, so neither glucose homeostasis nor oxidative status was formally characterized.

Generalization is limited in two further respects. The first is the use of male mice of a single outbred stock (Slc:ICR) at a single sucrose concentration. Because the allele frequencies of an outbred colony are undefined and differ between colonies and between facilities [44,45], Slc:ICR should not be regarded as a defined model of human population genetic structure, and testing whether the present findings depend on genetic background would require several inbred strains or a purpose-built heterogeneous stock. The timing of the pubertal androgen rise is likewise strain-dependent, and the trajectory against which the exposure window was set was measured in C57BL/6 and CD-1 mice [22]. The second is species: the relative reliance on the HDL/SR-BI pathway for steroidogenic Chol supply is greater in rodents than in humans. Extrapolation to humans should accordingly be made with caution.

## 5. Conclusions

In male Slc:ICR mice, free access to a 10% (w/v) sucrose solution from 4 to 8 weeks of age produced a cholesterol-predominant rather than a triglyceride-predominant lipid response. Despite transcriptional activation of the hepatic lipogenic program, triglycerides did not accumulate, and cholesterol rose in both the liver and the circulation, the increase being confined to the non-HDL fraction. Plasma testosterone was higher in the sucrose group, as was testicular Chol content under the reported analysis, while steroidogenic enzyme protein levels were unchanged; these observations are associated with one another, and the present data do not establish that the higher circulating testosterone arose from increased steroidogenesis rather than from altered clearance, binding or secretion. All measurements were made after a 16 h fast during which the sucrose solution had been replaced with water, and therefore describe the post-withdrawal state. The exposure provided approximately 25% of the total energy intake, and this was accompanied by a comparable fall in solid-food intake. Therefore, the present study focuses on the effect of sucrose taken as a beverage in combination with that compensatory reduction, rather than on the effect of sucrose alone. These changes occurred without any difference in body weight, body-weight gain, total energy intake, energy efficiency or blood glucose; peritesticular fat mass and blood β-hydroxybutyrate were, however, both higher in the sucrose group, and insulin, insulin resistance, oxidative stress and inflammatory markers were not assessed. These findings are stated for male Slc:ICR mice under the conditions described, and are not extended to other strains, to females, or to humans.

## Figures and Tables

**Table 1:** Primer sequences used in this study.

Gene Symbol	Primer F Sequence	Primer R Sequence
*Abca1*	GGGCATCTCCAGTTATGGCA	GTCTCCCTGGATTCTGGGTC
*Abcg5*	TCCTACAGCGTCAGCAACC	CAAACACCTCCCCTTCCAGG
*Acaca*	GCCGCCAGCCTGAGTTCTT	GGGAGCCCTCCTTCTCTTCT
*Actb*	TCTTTGCAGCTCCTTCGTTG	ACGATGGAGGGGAATACAGC
*Cyp11a1*	TCCTTTGAGTCCATCAGCAG	TGGGTGTACTCATCAGCTTTATTG
*Cyp17a1*	GCACAATCCTGAGGTGAAGAGG	ACTCTCCAATGCTGGAGTCAA
*Cyp19a1*	ATTTTCGCTGAGAGACGTGG	GATTGCTGCTTCGACCTCTG
*Fasn*	AGATGGAAGGCTGGGCTCTA	CCTCTGAACCACTCACACCC
*Hmgcr*	TTGACGCTCTTGTGGAATGC	CTCTAGGACCAGCGACACAC
*Hprt1*	TTAGCGATGATGAACCAGGTT	TCCTTCATGACATCTCGAGCA
*Hsd3b1*	GCCTTCATCTTCTGCAGCTC	CTGCCAGTACTGCCTTCTCA
*Hsd17b3*	CCAGAATCTCATCCACTGCA	AAGAGGCCAGGGACGAAG
*Ldlr*	CCAAATTCACCTGCGCCTG	TCACCCTGGACTTGGTGG
*Lhcgr*	ACTGAAAACTCTGCCCTCCA	TGGCAGAATAAAGCGTCTCG
*Ppia*	CCGATGACGAGCCCTTGG	GAGCTCGAAAGTTTTCTGCTGT
*Rpl19*	CAATGCCAACTCCCGTCAGC	TCTCAGGCATCCGAGCATTG
*Scarb1*	ACGGACTCAGCAAGATCGAT	TTGTAGGTCAGCTTCATGGAC
*Scp2*	CCTGGGGTTGGGATAATCGG	AATGCCTTCTGGCCTGCTTC
*Srebf1*	GGGGCCTGACAGGTGAAATC	GGAGGCCAGAGAAGCAGAAG
*Srebf2*	TCCCAAAGAAGGAGAGAGGC	CAATGGCCTTCCTCAGAACG
*Star*	TCTGCTTGGTTCTCAACTGG	ACTTCGTCCCCGTTCTCCT
*Stard3*	GTTGTGTCCCCCAGGGACTT	GTGGATAAGGTACCGAGGCA
*Tbp*	CCTATCACTCCTGCCACACC	ATGACTGCAGCAAATCGCTTG

Primers for *Srebf1* target transcript variant 3 (NM_001358314.1), which encodes SREBP-1c.

## Data Availability

Raw data supporting the conclusions of this study are available upon request from the authors.

## Supplementary Materials

The following supporting information can be downloaded at: https://www.mdpi.com/article/10.3390/nu18213481/s1, Figure S1: Correlation between testicular triglyceride (TG) and cholesterol (Chol) content; Figure S2: Reference gene primer validation; Figure S3: Effects of 10% sucrose solution intake on testicular steroidogenic enzyme protein expression in pubertal male mice; Table S1: Sample size for every reported endpoint; Table S2: Cq values of candidate reference genes in liver and testis; Table S3: Rodent studies of sugar exposure and circulating testosterone; Table S4: Sensitivity analysis with all available animals retained; Supplementary Methods: primer validation, selection of reference genes, and the cauda epididymal sperm count.

## Abbreviations

The following abbreviations are used in this manuscript:

ACC	Acetyl-CoA carboxylase
AMPK	AMP-activated protein kinase
BHB	β-hydroxybutyrate
BW	Body weight
Chol	Cholesterol
CPT1	Carnitine palmitoyltransferase 1
DNL	de novo lipogenesis
ELISA	Enzyme-linked immunosorbent assay
HDL	High-density lipoprotein
HDL-C	HDL-cholesterol
ICR	Institute of Cancer Research
IQR	Interquartile range
LDL	Low-density lipoprotein
LH	Luteinizing hormone
non-HDL-C	non-HDL-cholesterol
PBS	Phosphate-buffered saline
qPCR	Quantitative polymerase chain reaction
SEM	Standard error of the mean
SR-BI	Scavenger receptor class B type I
SREBP	Sterol regulatory element-binding protein
SSB	Sugar-sweetened beverage(s)
TG	Triglyceride
