# Supplementary material for: Low-Concentration Sucrose Intake During Puberty Alters Cholesterol Metabolism and Circulating Testosterone Levels in Male Mice
Source: Nutrients. 2026 Oct 23;18(21):3481. doi: 10.3390/nu18213481 (PMC13764763; doi:10.3390/nu18213481)
Supplement: Supplementary file 1 [file nutrients-18-03481-s001.zip › nutrients-4584770-supplementary.pdf]

Supplementary Figures and Tables

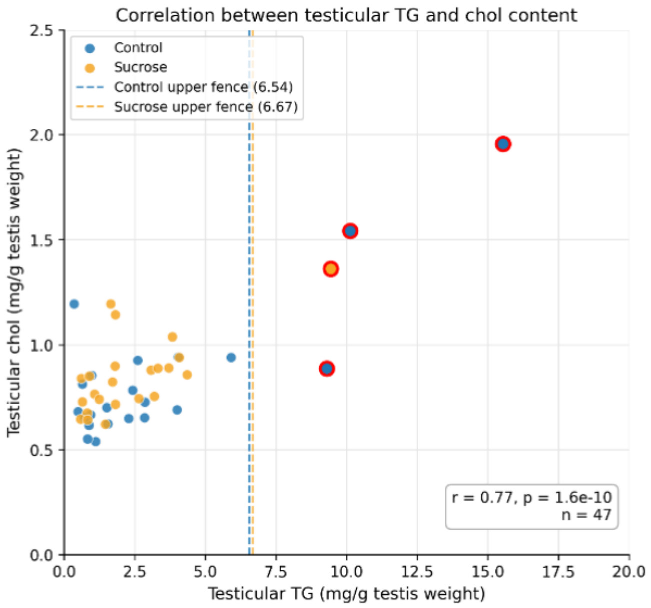

Supplementary Figure S1. Correlation between testicular triglyceride (TG) and cholesterol (Chol) content. TG and Chol content were strongly correlated across all animals (Pearson r = 0.78, $p < 0.0001$, n = 47). Extracts with a TG value exceeding the group-specific Tukey outlier threshold (1.5 × interquartile range; dashed lines) were considered contaminated by peritesticular adipose tissue and excluded from the testicular Chol analysis in Fig. 6a (red-outlined points; Control, n = 3; Sucrose, n = 1).

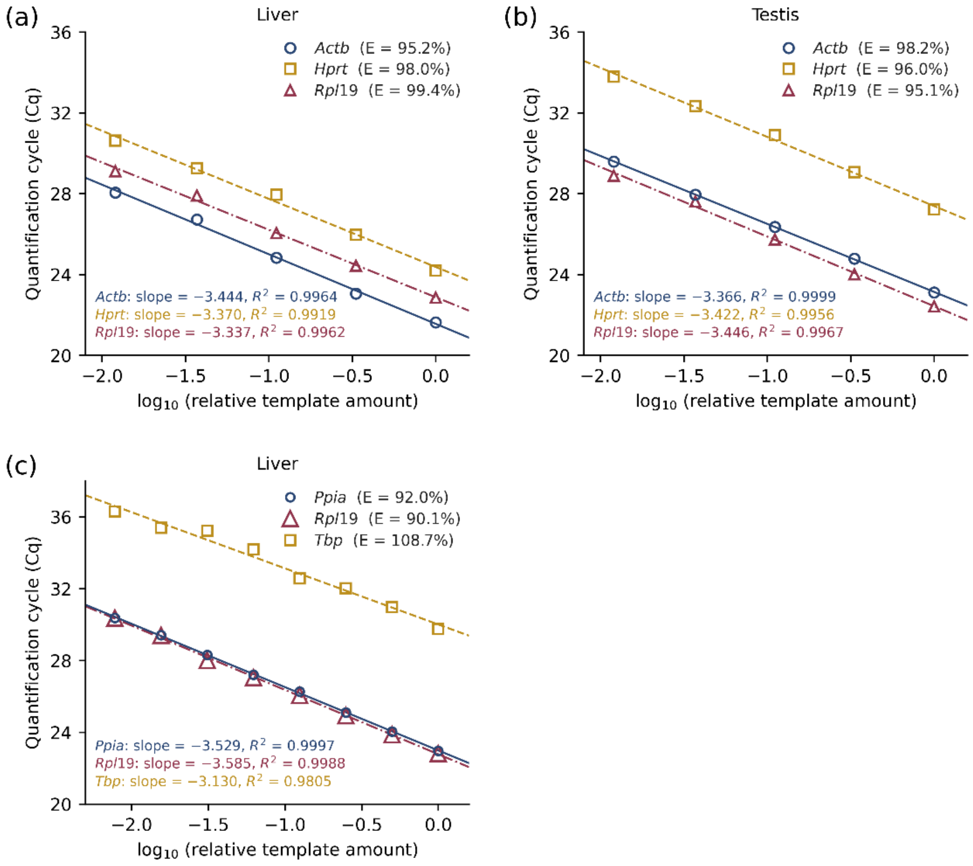

Supplementary Figure S2. Reference gene primer validation. (A) Standard curves obtained from a three-fold dilution series of pooled liver cDNA. Cq values were plotted against $\log_{10}$ of the relative template amount; lines indicate the linear regression fit for each gene (*Actb*: slope = −3.444, R^2 = 0.9964, E = 95.2%;

Hprt1: slope = −3.370, R^2 = 0.9919, E = 98.0%; Rpl19: slope = −3.337, R^2 = 0.9962, E = 99.4%). (B) Standard curves obtained from a three-fold dilution series of pooled testis cDNA (*Actb*: slope = −3.366, R^2 = 0.9999, E = 98.2%; *Hprt1*: slope = −3.422, R^2 = 0.9956, E = 96.0%; *Rpl19*: slope = −3.446, R^2 = 0.9967, E = 95.1%). No amplification was detected in the no-template controls, which are therefore not plotted. (c) Standard curves for the additionally evaluated hepatic reference genes, obtained from a two-fold dilution series of pooled liver cDNA (Ppia: slope = −3.529, R^2 = 0.9997, E = 92.0%; Rpl19: slope = −3.585, R^2 = 0.9988, E = 90.1%; Tbp: slope = −3.130, R^2 = 0.9805, E = 108.7%). Points are the means of replicate wells.

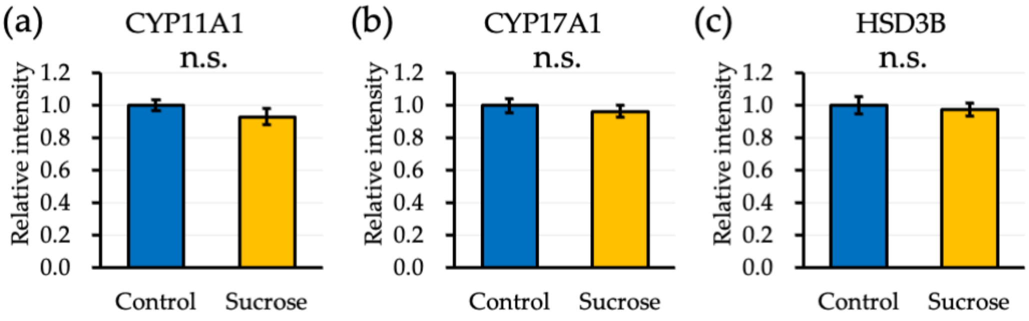

Supplementary Figure S3. Effects of 10% sucrose solution intake on testicular steroidogenic enzyme protein expression in pubertal male mice. Relative protein expression levels of CYP11A1 (a), CYP17A1 (b), and HSD3B (c) in Control and Sucrose-fed mice, normalized to a loading control and expressed relative to Control (= 1), as determined by Western blotting. Data are presented as mean ± SEM; n.s., not significant (Student's t-test, Control n = 24, Sucrose n = 23).

Supplementary Tables

Supplementary Table S1. Sample size for every reported endpoint.

Animals were allocated 24 per group. One sucrose-group animal was removed during the study for signs of poor health, giving 24 (C) and 23 (S) as the maximum available for any endpoint. Values below give the number contributing to each panel after the exclusions stated in the footnotes.

Panel	Endpoint	n (C / S)	Note
Table 2	Body weight, intake, organ weights	24 / 23	—
Figure 1a	Hepatic triglyceride	24 / 23	—
Figure 1b	Hepatic cholesterol	24 / 23	—
Figure 1c	Plasma triglyceride	13 / 12	h
Figure 1d	Plasma total cholesterol	13 / 12	h
Figure 1e	Plasma HDL-cholesterol	13 / 12	h
Figure 1f	Plasma non-HDL-cholesterol	13 / 12	h
Figure 2a	Hepatic *Srebf2*	24 / 23	—
Figure 2b	Hepatic *Hmgcr*	24 / 23	—
Figure 2c	Hepatic *Ldlr*	21 / 22	o
Figure 2d	Hepatic *Abca1*	22 / 22	o
Figure 2e	Hepatic *Abcg5*	21 / 23	o
Figure 2f	Hepatic *Scarb1*	23 / 23	o
Figure 3a	Hepatic *Acaca*	23 / 22	o
Figure 3b	Hepatic *Fasn*	22 / 21	o
Figure 3c	Hepatic *Srebf1*	22 / 23	o
Figure 3e	Hepatic ACC1 / β-actin	23 / 23	o
Figure 3f	Hepatic phospho-ACC1 / ACC1	23 / 22	o
Figure 3g	Hepatic ACC2 / β-actin	23 / 23	o

Figure 3h	Hepatic phospho-ACC2 / ACC2	24 / 23	—
Figure 3i	Hepatic AMPKα / β-actin	22 / 23	o
Figure 3j	Hepatic phospho-AMPKα / AMPKα	22 / 23	o
Figure 4a	Testicular *Scarb1*	23 / 23	o
Figure 4b	Testicular *Ldlr*	21 / 23	o
Figure 4c	Testicular *Hmgcr*	23 / 21	o
Figure 5a	Testicular *Star*	24 / 22	o
Figure 5b	Testicular *Scp2*	22 / 21	o
Figure 5c	Testicular *Stard3*	20 / 23	o
Figure 5d	Testicular *Cyp11a1*	24 / 23	—
Figure 5e	Testicular *Hsd3b1*	23 / 23	o
Figure 5f	Testicular *Cyp17a1*	24 / 23	—
Figure 5g	Testicular *Hsd17b3*	22 / 23	o
Figure 5h	Testicular *Cyp19a1*	21 / 22	o
Figure 6a	Testicular cholesterol	21 / 22	t
Figure 6b	Intratesticular testosterone	19 / 18	e, o
Figure 6c	Plasma testosterone	17 / 14	e, o, †
Suppl. Methods	Cauda epididymal sperm count	24 / 19	o
Suppl. Fig. S3	Testicular CYP11A1, CYP17A1, HSD3B1	24 / 23	—

— no exclusions; all available samples analyzed.

h hemolyzed plasma samples excluded from the enzymatic lipid assays (Methods 2.3).

o statistical outliers excluded by Tukey's method (1.5 × IQR) applied separately within each group (Methods 2.7).

t extracts with a triglyceride value identified as an outlier, indicating contamination by peritesticular adipose tissue, excluded (Methods 2.3).

e samples whose $\%B/B_0$ fell outside the working range of the ELISA standard curve, so that no concentration could be interpolated (Methods 2.4). These numbered 4 control and 5 sucrose for plasma, and 4 control and 4 sucrose for testis.

† no plasma sample was available for one sucrose-group animal.

Supplementary Table S2. Cq values of candidate reference genes in liver and testis

Tissue	Reference gene	Control (mean Cq ± SD)	n	Sucrose (mean Cq ± SD)	n	ΔCq	p	geNorm M
Testis	*Actb*	21.66 ± 0.34	24	21.63 ± 0.30	23	−0.04	0.72	—
Liver	*Ppia*	23.04 ± 0.56	24	23.94 ± 1.13	23	0.90	0.002	0.650
Liver	*Rpl*19	22.51 ± 0.60	24	23.05 ± 0.98	23	0.54	0.028	0.655
Liver	*Tbp*	29.70 ± 0.57	24	30.48 ± 0.91	23	0.78	0.001	0.678
Liver	*Hprt1*	25.85 ± 1.09	22	26.59 ± 1.24	23	0.75	0.037	0.975
Liver	*Actb*	22.48 ± 0.85	24	23.65 ± 0.84	23	1.17	<0.001	1.044
Liver	NF2	22.77 ± 0.56	24	23.50 ± 1.05	23	0.72	0.006	—
Liver	NF	23.62 ± 0.52	24	24.43 ± 0.91	23	0.81	<0.001	—

Values are mean Cq ± SD. ΔCq is sucrose minus control. p values are from two-tailed Welch's t-tests. *Hprt1* n in liver is reduced by two excluded assay failures. Liver genes are listed in order of increasing geNorm M (lower M indicates greater stability); M was computed on the 45 animals with complete data for all five candidates. In testis, only *Actb* was evaluated, so no M value is available. NF2, the normalization factor adopted in this study (mean Cq of *Ppia* and *Rpl19*); NF, the three-gene normalization factor used previously

(*Actb*, *Hprt1* and *Rpl19*), computed from gene-centred Cq values and shown here for comparison. See Supplementary Methods.

Supplementary Table S3. Rodent studies of sugar exposure and circulating testosterone, ordered by the age at which the animals were assessed

Study	Species and strain	Sugar, route and concentration	Age at start	Duration	Age at assessment	Circulating testosterone
De León-Ramírez et al. [1]	Rat, Wistar	Sucrose, drinking solution, 30% (w/v)	21 days (weaning)	30 days	51 days	Increased
Present study	Mouse, Slc:ICR	Sucrose, drinking solution, 10% (w/v)	4 weeks (P28)	4 weeks	8 weeks (P55)	Increased
Medaglia et al. [2]	Rat, Wistar	Fructose, drinking solution, 20%	PND 30	30 days	PND 60	Increased
Adekunbi et al. [3]	Rat, Sprague–Dawley	Sucrose, drinking solution, 30% (w/v)	8 weeks	6 weeks	14 weeks	Reduced
Hsia et al. [4]	Rat	Fructose, solid diet, 65%	6 weeks	21 weeks	27 weeks	Reduced

Values are as reported in the cited studies. "Increased" and "Reduced" denote the direction of the change in serum or plasma testosterone in the sugar-exposed group relative to its control. The four published studies were all carried out in rats; the present study used mice, and the two species differ in the timing of the pubertal androgen rise, as set out in the Discussion. P, postnatal day; PND, postnatal day.

Supplementary Table S4. Sensitivity analysis: every panel reanalyzed with all available animals retained.

The reported analysis is the one in the manuscript. In the second analysis no animal was excluded as an outlier. Fold change is the ratio of group means relative to control = 1. The test for each analysis was selected by the cascade in Methods 2.7 (Shapiro–Wilk, then Levene's test, then Student's t-test, Welch's t-test or the Mann–Whitney U test), so the test may differ between the two columns. Test: S, Student's t-test; W, Welch's t-test; MW, Mann–Whitney U test. Panels in bold are those whose conclusion changes at $\alpha = 0.05$.

Panel	Endpoint	As reported				All animals retained			
		n (C / S)	Fold	Test	p	n (C / S)	Fold	Test	p
Figure 2a	Hepatic *Srebf2*	24 / 23	1.05	MW	0.238	24 / 23	1.05	MW	0.238
Figure 2b	Hepatic *Hmgcr*	24 / 23	1.34	MW	0.042	24 / 23	1.34	MW	0.042
Figure 2c	Hepatic *Ldlr*	21 / 22	1.13	S	0.232	24 / 23	1.18	MW	0.157
Figure 2d	Hepatic *Abca1*	22 / 22	1.07	MW	0.069	24 / 23	1.17	MW	0.057
Figure 2e	Hepatic *Abcg5*	21 / 23	1.71	W	<0.0001	24 / 23	1.72	S	<0.0001
Figure 2f	Hepatic *Scarb1*	23 / 23	0.98	S	0.851	24 / 23	1.01	S	0.897
Figure 3a	Hepatic *Acaca*	23 / 22	1.58	W	0.0003	24 / 23	1.79	MW	0.0001
Figure 3b	Hepatic *Fasn*	22 / 21	1.33	S	0.005	24 / 23	1.54	MW	0.007
Figure 3c	Hepatic *Srebf1*	22 / 23	2.32	MW	0.025	24 / 23	2.51	MW	0.010

Figure 3e	Hepatic ACC1 / β-actin	23 / 23	1.36	MW	0.008	24 / 23	1.26	MW	0.021
Figure 3f	Hepatic phospho-ACC1 / ACC1	23 / 22	1.45	S	0.002	24 / 23	1.37	MW	0.010
Figure 3g	Hepatic ACC2 / β-actin	23 / 23	1.05	S	0.646	24 / 23	0.97	MW	0.694
Figure 3h	Hepatic phospho-ACC2 / ACC2	24 / 23	1.51	S	<0.0001	24 / 23	1.51	S	<0.0001
Figure 3i	Hepatic AMPKα / β-actin	22 / 23	0.94	S	0.069	24 / 23	0.94	S	0.091
Figure 3j	Hepatic phospho-AMPKα / AMPKα	22 / 23	1.02	MW	0.794	24 / 23	0.93	MW	0.476
Figure 4a	Testicular *Scarb1*	23 / 23	1.15	S	0.005	24 / 23	1.12	S	0.027
Figure 4b	Testicular *Ldlr*	21 / 23	1.41	MW	0.411	24 / 23	0.87	MW	0.992
Figure 4c	Testicular *Hmgcr*	23 / 21	1.15	MW	0.166	24 / 23	1.36	MW	0.128
Figure 5a	Testicular *Star*	24 / 22	1.00	MW	0.817	24 / 23	1.05	MW	0.632
Figure 5b	Testicular *Scp2*	22 / 21	1.10	MW	0.799	24 / 23	1.01	MW	0.823
Figure 5c	Testicular *Stard3*	20 / 23	1.39	W	<0.0001	24 / 23	1.26	MW	0.0002
Figure 5d	Testicular *Cyp11a1*	24 / 23	1.05	S	0.631	24 / 23	1.05	S	0.631
Figure 5e†	Testicular *Hsd3b1*	23 / 23	1.22	S	0.0010	24 / 23	0.37	MW	0.006
Figure 5f	Testicular *Cyp17a1*	24 / 23	1.28	S	0.0005	24 / 23	1.28	S	0.0005
Figure 5g	Testicular *Hsd17b3*	22 / 23	1.39	S	<0.0001	24 / 23	1.32	S	0.0002
Figure 5h	Testicular *Cyp19a1*	21 / 22	1.12	S	0.177	24 / 23	1.01	MW	0.840
**Figure 6a‡**	**Testicular cholesterol**	**21 / 22**	**1.13**	**MW**	**0.032**	**24 / 23**	**1.03**	**MW**	**0.123**
Figure 6b§	Intratesticular testosterone	19 / 18	1.32	MW	0.230	20 / 19	1.33	MW	0.244
Figure 6c§	Plasma testosterone	17 / 14	1.39	MW	0.010	20 / 17	1.12	MW	0.040
Suppl. Methods	Cauda epididymal sperm count	24 / 19	1.26	S	0.020	24 / 23	1.36	S	0.014

† Testicular Hsd3b1. One control animal has a ΔCq 5.4 cycles below the next value, corresponding to approximately 53 times the control median relative expression. With that animal retained the ratio of group means is dominated by it and formally reverses direction, whereas the ratio of group medians (1.13) and the rank-based test (p = 0.006) both retain the direction and the significance reported in the manuscript.

‡ Testicular cholesterol. The exclusion applied here was not an outlier rule applied to the cholesterol values. Extracts were excluded when their triglyceride content was an outlier, which identifies carry-over of peritesticular adipose tissue and is therefore independent of the cholesterol value being tested. The second analysis retains those extracts.

§ Testosterone panels. Samples whose $\%B/B_0$ fell outside the working range of the ELISA standard curve were not assigned a concentration and so cannot be restored; the second analysis retains only the samples excluded as statistical outliers.

Supplementary Figure S3 (testicular CYP11A1, CYP17A1 and HSD3B protein) is not listed because no animal was excluded from it; all 24 and 23 were analyzed as reported. Outliers were identified on the quantity to which the cascade was applied: the ΔCq for the hepatic transcripts, and the relative value for the testicular transcripts, the immunoblot ratios and the remaining endpoints.

Supplementary Methods

Primer validation

Amplification efficiencies of the primer pairs for the reference genes were determined from standard curves generated with serial dilutions of pooled cDNA. For *Actb*, *Hprt1* and *Rpl19*, a three-fold dilution series was used (relative template amount 1 to 0.012, five points) in each tissue. Cq values were plotted against log10 of the relative template amount and fitted by linear regression, and amplification efficiency (E) was calculated as $E = 10^{(-1/\text{slope})} - 1$. In liver, the slopes were −3.444 for *Actb* ($y = -3.444x + 21.56$, $R^2 = 0.9964$), −3.370 for *Hprt1* ($y = -3.370x + 24.37$, $R^2 = 0.9919$), and −3.337 for *Rpl19* ($y = -3.337x + 22.87$, $R^2 = 0.9962$), corresponding to efficiencies of 95.2%, 98.0% and 99.4%, respectively. In testis, the slopes were −3.366 for *Actb* ($y = -3.366x + 23.14$, $R^2 = 0.9999$),

−3.422 for *Hprt1* (y = −3.422x + 27.39, R^2 = 0.9956), and −3.446 for *Rpl19* (y = −3.446x + 22.43, R^2 = 0.9967), corresponding to efficiencies of 98.2%, 96.0% and 95.1%, respectively.

Ppia and *Tbp* were subsequently introduced as additional hepatic reference genes, and *Rpl19* was re-determined alongside them, using a two-fold dilution series of pooled liver cDNA (relative template amount 1 to 0.008, eight points). The slopes were −3.529 for *Ppia* (y = −3.529x + 22.99, R^2 = 0.9997), −3.585 for *Rpl19* (y = −3.585x + 22.78, R^2 = 0.9988), and −3.130 for *Tbp* (y = −3.130x + 30.01, R^2 = 0.9805), corresponding to efficiencies of 92.0%, 90.1% and 108.7%, respectively.

All efficiencies fell within the range recommended for the comparative Cq method, and the two genes used to derive the hepatic normalization factor (*Ppia* and *Rpl19*) differed in efficiency by 1.9 percentage points. Standard curves are shown in Supplementary Figure S2. No amplification was detected in no-template controls for any assay.

Selection of reference genes

Because the suitability of a reference gene is tissue-dependent, stability was assessed separately for liver and testis. In each tissue, Cq values were compared between the control and sucrose groups before any target gene was analyzed.

In testis, *Actb* did not differ between groups (−0.04 Cq, $p = 0.72$) and showed low within-group variability (SD 0.34 and 0.30 for control and sucrose, respectively). It was therefore used as the sole reference gene, and ΔCq was calculated as Cq(target) − Cq(Actb).

In liver, none of the five candidates was unchanged between groups: *Actb* differed by +1.17 Cq ($p < 0.001$), *Ppia* by +0.90 Cq ($p = 0.002$), *Tbp* by +0.78 Cq ($p = 0.001$), *Hprt1* by +0.75 Cq ($p = 0.037$) and *Rpl19* by +0.54 Cq ($p = 0.028$). Selection was therefore based on expression stability rather than on the absence of a group difference. The stability of all five candidates was evaluated by geNorm [5], which ranks genes by the average pairwise variation of their log ratios with the remaining candidates. Stability values (M) were 0.650 for *Ppia*, 0.655 for *Rpl19*, 0.678 for *Tbp*, 0.975 for *Hprt1* and 1.044 for *Actb*; the pairwise variation on moving from two to three reference genes was V(2/3) = 0.133, below the conventional 0.15 threshold. Two reference genes were therefore used, and the normalization factor (NF2) was defined as the mean Cq of *Ppia* and *Rpl19*. ΔCq was calculated as Cq(target) − NF2. Both genes were measured successfully in every animal, so neither centering nor imputation was required.

Because M is computed from the standard deviations of pairwise log ratios between candidate genes, any component of variation shared by all transcripts within a sample cancels and does not contribute to M; the ranking above is therefore unaffected by whether the shift common to the hepatic reference genes is technical or biological in origin. A shift of this kind is, however, not removed by normalization to any reference gene. This residual uncertainty applies equally to every hepatic transcript reported here and does not affect the direction of the between-group comparisons.

Hprt1 measurements for two control animals were excluded as assay failures, as their Cq values (20.60 and 20.46) fell more than 4 Cq below those of all other animals (25.07–27.82), a pattern consistent with contamination rather than with biological variation. These two animals are excluded from the *Hprt1* row of Supplementary Table S1 and from the geNorm computation, which used the 45 animals with complete data for all five candidates. The three-gene normalization factor used in the original analysis (*Actb*, *Hprt1* and *Rpl19*) is shown in Supplementary Table S1 for comparison; it was computed from gene-centred Cq values so that animals lacking *Hprt1* were not biased by the approximately 3.4 Cq offset between *Hprt1* and the other two genes.

Cauda epididymal sperm count

The sperm count was evaluated in the cauda epididymis. The tissue was incised and placed in 1× PBS, and the spermatozoa were allowed to swim out at room temperature for approximately 3 min. An equal volume of 4% paraformaldehyde (PFA; Nacalai Tesque) was then added to fix the

spermatozoa. The sperm count was determined using a hemocytometer (Cat. No. 177-112C; WATSON, Tokyo, Japan) and is expressed as the number of spermatozoa per gram of cauda epididymis.

The cauda epididymal sperm count was $88.3 \pm 6.0 \times 10^6$ per g in group C (n = 24) and $110.9 \pm 7.2 \times 10^6$ per g in group S (n = 19); p = 0.0199, Student's t-test, after the exclusion of statistical outliers by Tukey's method applied separately within each group. Sperm motility, morphology and viability, testicular sperm counts, and histological evaluation of the testes and epididymides were not performed. This measurement is reported for completeness and does not constitute an assessment of reproductive function.

Reference

1. De Leon-Ramirez, Y.M.; Lara-Garcia, M.; Pacheco, P.; Lara-Garcia, O.; Martinez-Gomez, M.; Cuevas-Romero, E.; Rodriguez-Antolin, J.; Nicolas-Toledo, L. Histomorphological testicular changes and decrease in the sperm count in pubertal rats induced by a high-sugar diet. *Ann Anat* **2021**, *235*, 151678, doi:10.1016/j.aanat.2021.151678.
2. Medaglia, D.S.A.; Vieira, H.R.; Silveira, S.D.S.; Siervo, G.; Marcon, M.; Mathias, P.C.F.; Fernandes, G.S.A. High-fructose diet during puberty alters the sperm parameters, testosterone concentration, and histopathology of testes and epididymis in adult Wistar rats. *J Dev Orig Health Dis* **2022**, *13*, 20-27, doi:10.1017/S2040174420001385.
3. Adekunbi, D.A.; Ogunsola, O.A.; Oyelowo, O.T.; Aluko, E.O.; Popoola, A.A.; Akinboboye, O.O. Consumption of high sucrose and/or high salt diet alters sperm function in male Sprague–Dawley rats. *Egyptian Journal of Basic and Applied Sciences* **2016**, *3*, 194-201, doi:10.1016/j.ejbas.2016.03.003.
4. Hsia, S.M.; Chiang, Y.F.; Chen, H.Y.; Ali, M.; Wang, P.S.; Wang, K.L. Effect of High-Fructose Diet-Induced Metabolic Syndrome on the Pituitary-Gonadal Axis in Male Rats. *Biomedicines* **2022**, *10*, doi:10.3390/biomedicines10123009.
5. Vandesompele, J.; De Preter, K.; Pattyn, F.; Poppe, B.; Van Roy, N.; De Paepe, A.; Speleman, F. Accurate normalization of real-time quantitative RT-PCR data by geometric averaging of multiple internal control genes. *Genome Biol* **2002**, *3*, RESEARCH0034, doi:10.1186/gb-2002-3-7-research0034.
